# Population Attributable Fraction of Early Age of Onset of Alcohol Use in Alcohol Abuse and Dependence: A 3-Year Follow-Up Study in University Students

**DOI:** 10.3390/ijerph17062159

**Published:** 2020-03-24

**Authors:** Francisco Caamano-Isorna, Amy Adkins, Fazil Aliev, Lucía Moure-Rodríguez, Danielle M. Dick

**Affiliations:** 1 Department of Psychology, Virginia Commonwealth University, Richmond, VA 23284, USA; francisco.caamano@usc.es (F.C.-I.); adkinsae@vcu.edu (A.A.); faliev@vcu.edu (F.A.); ddick@vcu.edu (D.M.D.); 2 CIBER de Epidemiología y Salud Pública (CIBERESP), Department of Public Health. Universidade de Santiago de Compostela, 15706 Santiago de Compostela, Spain; 3 College Behavioral and Emotional Health Institute, Virginia Commonwealth University, Richmond, VA 23284, USA; 4 Faculty of Business, Karabuk University, 78050 Karabuk, Turkey; 5 Department of Innovation and Research. Complexo Hospitalario Universitario de Ourense, 32005 Ourense, Spain; 6 Department of Human and Molecular Genetics, Virginia Commonwealth University, Richmond, VA 23284, USA

**Keywords:** alcohol drinking in college, age of onset, risk factors, cohort

## Abstract

Background: we aimed to determine the risk factors and associated population attributable fractions (PAFs) for the age of onset of alcohol use and also to identify protective factors. Methods: we analyzed follow-up data collected between autumn 2011 and spring 2016 (*n* = 5170) from the first two cohorts (2011, 2012) of the Spit for Science^TM^ project. The dependent variables were alcohol abuse and dependence, and the independent variables were age of drinking onset, residence, ethnicity, religiosity, sexual orientation and work status. We determined the odds ratios (OR) using multilevel logistic regression for repeated measures in SPSSv.20. Results: the early onset of alcohol use was associated with an increased risk of alcohol abuse and dependence among females (OR = 14.98; OR = 11.83) and males (OR = 7.41; OR = 6.24). The PAFs for the early onset of alcohol use in alcohol abuse and dependence were respectively 80.9% and 71.7% in females and 71.0% and 63.5% in males. Among females, being white (OR = 1.58; OR = 1.51), living off-campus (OR = 1.73; OR = 2.76) and working full-time (OR = 1.69; OR = 1.78) were also risk factors. Strong religious beliefs were found to protect males from alcohol abuse (OR = 0.58), while same-gender sexual orientation increased the risk among females (OR = 2.09). Conclusion: delaying the age of onset by one year would reduce alcohol abuse among young adults.

## 1. Introduction

According to the World Health Organization [1,2], alcohol consumption among adolescents and young adults constitutes a public health problem worldwide, especially in the US and Europe. Due to the fact that the adolescent brain is still developing, it is vulnerable to the neurotoxic effects of alcohol. Unsurprisingly, alcohol use among young adults has been associated with social negative effects such as poorer academic achievement [3,4], greater consumption of prescription drugs [5,6,7] and injuries [8,9], and it also has psychological and physical negative consequences [10,11,12]. 

The high prevalence of risky habits among adolescents and young adults has been highlighted in several studies [13], including high levels of risky alcohol use, understood as consumption that increases the risk of adverse consequences [14,15]. In addition, university students appear to practice more dangerous alcohol consumption than their non-university peers [16,17,18]. The practice of binge drinking is common among this population group, an especially dangerous pattern that has been associated with an increased risk of negative consequences in the short and medium term [19].

Many risk factors have been identified in relation to risky drinking habits among young adults, including genetics, sociodemographic characteristics—such as gender, age or ethnicity, substance-related factors such as age of onset or other substance use, living circumstances—for example, living on campus or at the family home, and university characteristics—for example academic year [15,20,21,22,23]. To date, the age-related distribution of prevalence rates peaked at around 21 years in the US [24] and at between 19 and 24 years in Europe, depending on the country and gender, before declining at later ages [17,21,25]. 

According to the Diagnostic and Statistical Manual of Mental Disorders, 4th Edition (DSM-IV), alcohol abuse and dependence on alcohol are maladaptive drinking patterns that lead to significant distress or impairment. Alcohol abuse (AA) implies the manifestation of at least one of four criteria and non-compliance with criteria for alcohol dependence (AD), while alcohol dependence implies the manifestation of at least three of seven different criteria, in both cases within a 12-month period [26]. At present DSM-V integrates these two disorders, alcohol abuse and alcohol dependence, into a single disorder called alcohol use disorder (AUD), with mild, moderate, and severe sub-classifications. Regarding alcohol abuse and alcohol dependency among university students in the US, a study by Knight and colleagues [27] reported the prevalence of abusive consumption among undergraduates to be 31% and dependence, 6% (one in five binge drinkers was classified as alcohol dependent). Heather et al. reported similar data in a study conducted at seven UK universities [28], with higher prevalence in young people than in older age groups [29]. 

Given the high prevalence of heavy drinking and alcohol-related problems in the university environment, we decided to examine the corresponding data in a sample of university students. We used data from the Spit for Science project [30], an ongoing university-wide research project carried out in the US, which longitudinally assesses genetic and environmental influences on substance use and psychiatric disorders in a representative sample from a large urban university. The objectives of the present study were to report alcohol abuse and dependence, to identify explanatory factors for alcohol abuse and dependence, and to determine the population attributable fractions (PAFs) for the potentially modifiable risk factors among university students.

## 2. Materials and Methods

### 2.1. Design, Population and Sample 

The data analysed were obtained in a cohort study among university students between the first and the last waves of the Spit for Science™ study. The present analysis includes follow-up data on students in the first two cohorts (2011, 2012), collected between autumn 2011 and spring 2016. All incoming first year students aged 18 years or older were invited to participate in the study (*N* = 7049; 3113 women and 2057 men). The participation rate at the beginning of the study was 73.3% (*N* = 5170). The University Institutional Review Board approved all study procedures, and informed consent was obtained from all participants. Subjects were informed that participation was voluntary and confidential, and that they could withdraw from the study at any time.

### 2.2. Data Collection Procedure

The study data were collected and managed with REDCap (Research Electronic Data Capture), electronic data capture tools hosted at a mid-Atlantic public university [31]. REDCap is a secure, web-based application designed to support data capture for research studies, providing (1) an intuitive interface for validated data entry, (2) audit trails for tracking data manipulation and export procedures, (3) automated export procedures for seamless data downloads to common statistical packages and (4) procedures for importing data from external sources. Subjects received $10 and a t-shirt for participating in the study. Participants who were enrolled in autumn were invited to complete follow-up surveys each spring, beginning in their first year at university, while those enrolled in the spring were invited to complete follow-up surveys, beginning in the spring of their first year. During the spring semester of the first year, an invitation to participate in the study was also sent to individuals who had not joined in autumn. The new spring survey asked participants to retrospectively report on the items from the autumn survey, as well as to report on current items. Participants in both cohorts thus had four opportunities to complete the survey (autumn assessment and three spring follow-up assessments). Further details regarding data collection are reported by Dick et al. 2014 [28]. The authors agree to make freely available the questionnaire upon reasonable request.

### 2.3. Definition of Variables 

#### 2.3.1. Dependent Variables

Alcohol abuse and alcohol dependence. These variables were assessed using items adapted from the Semi-Structured Assessment for the Genetics of Alcoholism (SSAGA) instrument [32]. Diagnoses and symptoms for each of the following are available for all waves. The fourth version of the DSM (DSM-IV) was used, because it was in force at the time of the study. For the same reason, the variables regarding alcohol consumption used are alcohol abuse and alcohol dependence, as defined in the DSM-IV. The strict criteria of the DSMIV were followed in order to classify abuse or dependence. The DSMIV establishes dependence regardless of alcohol abuse, and therefore there may be some cases in which subjects were identified as alcohol dependent, even though they did not meet criteria for alcohol abuse. Non-drinkers were considered “non-abusers and non-dependent”.

#### 2.3.2. Independent Variables

Subjects were questioned about the age of onset of alcohol use, defined as the first time that they consumed alcohol, understood as a regular consumption and not only trying alcohol or having taken a drink from another person’s alcoholic beverage. This variable was measured using an item of the Substance Abuse and Mental Health Services 2013 questionnaire [33]. Five categories of this variable were defined: older than 18 years, 18 years old, 17 years old, 16 years old, younger than 16 years. Cannabis consumption was measured in each survey with the question “Have you consumed cannabis in the last 12 months? Yes/No”. This variable was also measured according to the Substance Abuse and Mental Health Services 2013 questionnaire [31]. 

Subjects were also asked about their religious beliefs. Religiosity was measured using a scale developed and validated by Kendler et al. [34] The scale combined/adapted items from the National Comorbidity survey, Strayhorn’s religiousness scale and a Gallup Poll. A score from 2 to 8 was generated from the questionnaire. The results of this score were coded in tertiles, with the third tertile being 33% of the subjects with the highest degree of religiosity.

Finally, several socio-demographic and personal characteristic variables were considered: gender, ethnicity, sexual orientation (heterosexual/homosexual/bisexual), place of residence (parental home/residence hall/off-campus housing) and work status (currently not working/part-time/full-time). The three last variables were measured in each survey. 

### 2.4. Statistical Analysis

We used multilevel logistic regression for repeated measures to obtain adjusted odds ratios (OR) for independent variables from the AA and AD models. Confidence intervals of 95% (95% CI) were calculated. These models are more flexible than traditional models, and therefore allowed us to work with correlated data, with a dependency structure. The same subject was measured several times, and the responses were strongly correlated. The cohort was considered a random variable. 

Maximal models, including all theoretical independent variables, were generated. The maximal models were used to generate final models, which included all significant variables or non-significant variables when their exclusion changed the OR of other variables by more than 10%. Data were analyzed using generalized linear mixed models, in SPSS v.20 (SPSS Inc., Chicago, IL, USA) statistical software.

Finally, in order to determine the impact of the delay of onset of alcohol use, we calculated the population attributable fractions (PAFs) for alcohol abuse and alcohol dependence. In addition, although in recent years we have observed changes in the profiles of the drinkers [35], and as it would be unrealistic to assume that young people only start drinking alcohol at age 18 years, we also calculated the proportion of alcohol abuse and alcohol dependence that would theoretically be removed if different proportions of students (a third of students, a half of students, and all students) began consuming alcohol one year later. For all these calculations, we used the formulas proposed by Morgenstern and Bursic [36]. All analyses in the present study were performed from a gender perspective, i.e., men and women were analysed separately. This enabled us to determine the prevalence in each group, as well as identify the explanatory factors for each group, taking into account that these may differ in men and women.

## 3. Results

The characteristics of the samples of women and men are summarized in Table 1 and Table 2, respectively. Attrition of subjects occurred during the follow-up period. The age of onset of alcohol consumption differed significantly between both men and women throughout the follow-up, and the proportion of those who began to consume alcohol after age 18 years increased (from 19.9% to 32.2% in women and from 16.5% to 27.1% in men). Among women, there were also significant differences regarding ethnicity, decreasing from 48.9% of white women at the start of the study to 42.5% at the end of the follow-up. Finally, for both men and women there were statistically significant differences for cannabis use, only when taking into account the linear association with Χ^2^. Nevertheless, there were no clinically relevant differences between the initial sample and follow-up samples in the main variables, so that the representativeness of the initial sample was maintained throughout the follow-up, for both men and women. 

The rates of prevalence of alcohol abuse and alcohol dependence were always lower in women than in men, although the differences were not statistically significant. The rates of prevalence of alcohol abuse and alcohol dependence in each age group of women and men are shown in Table 3 and Table 4, respectively.

Multivariate logistic models show that the early onset of alcohol use is the main risk factor for alcohol abuse and alcohol dependence among both females (Odds Ratio [OR] = 14.98 & OR = 11.83) and males (OR = 7.41 & OR = 6.24), respectively (Table 5).

**Figure 1:**
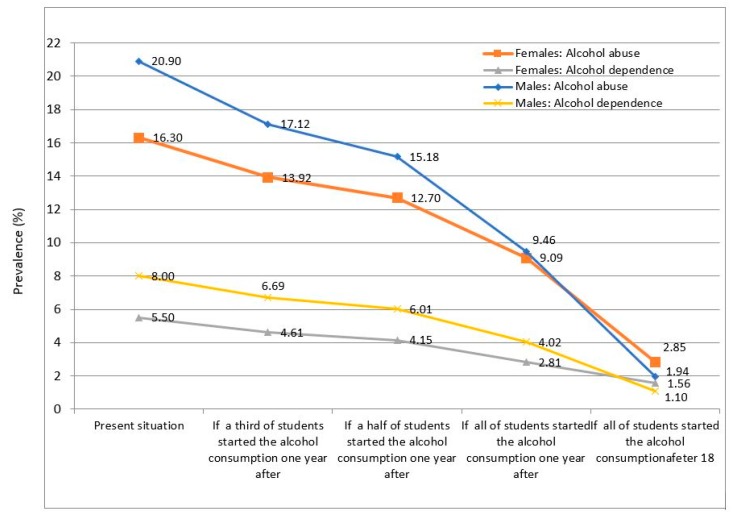
Estimated impact of the delay in the age of onset on alcohol abuse and dependence.

Table 6 shows the PAF of the early age of onset of alcohol abuse and alcohol dependence. Figure 1 shows the estimated prevalence of alcohol abuse and dependence, according to population attributable fractions. A delay in the age of onset of alcohol use would have a significant impact on both gender and outcome.

Regarding the sociodemographic variables among females, being white (OR = 1.58 & OR = 1.51), living off-campus (OR = 1.73 & OR = 2.76) and working full-time (OR = 1.69 & OR = 1.78) are also risk factors for alcohol abuse and alcohol dependence, respectively. In addition, holding strong religious beliefs was identified as a protective factor for alcohol abuse in males (OR = 0.58), and same-gender sexual orientation was found to be a risk factor for alcohol abuse among females (OR = 2.09). These data are summarized in Table 5.

## 4. Discussion

The findings indicate a high prevalence of alcohol abuse and alcohol dependence in the population of university students under study. Moreover, multivariate models showed that the early age of onset of drinking is the most important risk factor for both outcomes in both females and males. Population attributable fractions show that delaying the age of onset of alcohol use by just one year would reduce alcohol abuse among young people. Among women, being white, living off-campus and working full-time were also identified as risk factors for both alcohol abuse and dependence. Finally, in men holding strong religious beliefs protects them, while in women a same-gender orientation increases the risk for alcohol abuse only.

The rates of prevalence of alcohol abuse and alcohol dependence were always lower in women than men. Higher alcohol use or alcohol dependence among men than among women is common in many cultures [37,38]. However, these differences tend to disappear in young people [39,40]. Our findings show higher alcohol abuse and alcohol dependence among males, although the differences were not statistically significant. This lack of statistical significance in the differences may be due to the telescopic effect that consists on faster progression among women to alcohol abuse or dependency after alcohol initiation [41,42]. Following this reasoning, in a few years we could find higher figures of abuse or dependency in men, that would increase the differences between genders. However, it can also be explained by a narrowing in the differences between genders regarding alcohol consumption among the younger generations, more marked in countries where traditional gender roles have changed more [43].

Although the differences in alcohol dependence were not statistically significant, we observed a slight tendency for rates to increase in men over time, while in women the tendency tended to decrease. This is consistent with the earlier peak in women observed by some authors regarding alcohol consumption trends among university students [44].

Nevertheless, the rates of prevalence of alcohol abuse remained fairly constant during the three years of follow-up. Perhaps, in this case, the peak consumption has not been reached in either gender, an interpretation previously made by other authors with similar results [17,21]. It will be interesting to observe how these trends evolve in the coming years. 

The high prevalence rates observed in our study are similar to those reported by Knight in 2002 [25], although higher than those reported in other studies of university students [27,45]. However, the proportion of dependence and abuse in the adult population is usually lower [46,47]. This may be partly explained by the association between alcohol dependence and excessive alcohol consumption observed by some authors [41], and which is more frequent among young adults and university students [14,15], or by problems in the measurement of abuse and/or dependence among young adults previously referred to by some authors [27,48].

For both alcohol abuse and alcohol dependence, the multivariate logistic models show that the early onset of alcohol use is the main risk factor. The age of onset has classically been studied in relation to alcohol consumption and its consequences in different contexts and ages, with variable results [49,50]. In this regard, our findings are consistent with several scientific studies, showing this variable to be an important predictor for alcohol use or its negative consequences, and even specifically for abuse and dependence among young adults [45,51].

Bearing this in mind, we decided to assess how delaying the age of onset of alcohol use would affect the prevalence of alcohol abuse and dependence among the university students under study. The results showed important reductions in both conditions. In addition, delaying the age of onset of alcohol use until 18 years, an age at which in the US drinking is still illegal, suggests the importance of reinforcing compliance with current legislation, which could help reduce alcohol abuse and dependence among university students. It must be remembered that we did not adjust for some of the variables that previous authors have suggested may influence the age of onset [52,53], such as parental permissiveness or peer approval; however, adding other covariates would not imply an absence of the relationship found, and the calculations regarding the delay of the age of onset should not be greatly affected.

For both alcohol abuse and alcohol dependence, three variables acted as risk factors among women in the present study. First of all, being white increased the risk of alcohol abuse and dependence among female college students. The higher risk among white people has previously been observed in other studies related to onset of alcohol consumption, i.e., in alcohol use itself among university students, and specifically alcohol dependence [25,26,54]. It is difficult to establish an explanation for this finding. Skidmore et al. found in 2013 that European American college students were at increased risk for alcohol-related problems and suggests that this kind of problem may be more accepted among this group [55]. On the other hand, Delva et al. found that African American female students practice more protective behaviors [56]. According to Skidmore, parents’ attitudes regarding substance use may also be affecting these young women more than European Americans, who would not have such a close relationship with their families. The reality is that the scientific evidence regarding alcohol related problems differences based on ethnicity is mainly focused on adolescents or adults, but it is scarce among college population, as Clarke et al. remind us, so more research is need for been able to corroborate our results, and then assess possible explanations for them [57].

Living off-campus, outside of university residences and away from the parental home have also been shown to be risk factors for both alcohol abuse and alcohol dependence, although only in women. Similar results were observed in previous studies [21,40], possibly due to a stricter and more controlled home environment [58,59], mainly for young females. Moreover, the study cohorts included more females, and the lack of significance of this variable in males may be due to lower statistical power.

Finally, working full time also acted as a risk factor. Understanding this variable as an indicator of socioeconomic status suggests that lower socioeconomic resources increase the risk of alcohol abuse and alcohol dependence among female college students. The reported findings on this topic are variable [60]. Accessibility to alcohol is also closely related to economic income [21], which may also explain the association between full-time working status and the higher incidence of alcohol abuse and alcohol dependence observed in our study.

Alcohol abuse in some individuals may be a strategy for coping with social pressure and rejection; this is one of the causes most commonly used to explain the higher consumption and levels of alcohol abuse in homosexual and bisexual participants [61]. We also observed a higher risk of alcohol abuse among homosexual students, a finding that is consistent with those reported in the National Health Interview Survey and in other studies [62,63,64], although in the present study the effect was only maintained in women after adjusting for the remaining variables. One possible explanation for this, suggested by Lyons [65], is that access to some support resources is more limited among gay women than among gay men.

The present study showed that a high level of religiosity is a protective factor for alcohol abuse. Other studies have shown that subjects with higher levels of religiosity tend to have a more orderly life and behave more prudently [66,67]. Some authors have explained this effect as being due to the influence of peers, as well as the control/protection exercised by the religious community [68]. However, after the other variables were adjusted for, the effect was only maintained in male students, which has not been mentioned in previous studies. The fact that, in our study, female students presented significantly higher religiosity than males, could be influencing this lack of association.

We have identified seven main limitations to this study: 1) the study was carried out in a single large, urban public university, which should be taken into account when generalizing the results; 2) the data are part of a wider longitudinal study and, therefore, the data and measurements used were not specifically designed for the purposes of the present study; 3) selection was biased because of the loss of subjects at follow-up; however, despite the significant differences in three of the variables, we do not consider that there are any clinical differences between the initial samples and the follow-up samples, and we therefore consider that the representativeness of the initial sample remains during the whole follow-up, suggesting the absence of bias; 4) self-reported data may be skewed due to inconsistencies in personal feelings or memories; 5) asking participants in the new spring survey to retrospectively report on the items from the fall survey, as well as to report on current items, may be a methodological flaw, because collapsing baseline alcohol measures across current use and retrospective accounts at much later dates may generate inaccurate data. However, we believe that it is worth maximizing participation, despite the possible loss of some accuracy in the measures; and 6) the validity of the conclusions may be limited by the difficulty in differentiating between cause and effect. However, in this case the factors associated with alcohol abuse and alcohol dependence (i.e., age of onset of alcohol use, residence, ethnicity) are unlikely to vary during the period in which the dependent variable was measured. This implies that cross-sectional analysis is equally as useful as longitudinal analysis [69], and that our results may provide advice for young adults (other than the study participants) in the future, preventing them from acquiring abuse or dependence on alcohol; 7) the reward in the initial sample may be causing the participating subjects to have a specific profile related to the appetite for this reward, however it is a technique widely used and accepted in the scientific community, which aims to increase participation, so it is important to create powerful studies with solid results.

## 5. Conclusions 

The study findings have important practical implications. Encouraging individual predisposition to healthy behavior is important, especially in this group in which alcohol consumption is highly normalized. Social awareness about the negative effects of earlier alcohol consumption should be increased, leading to a greater perception of the risks involved. Knowing in as much detail as possible the population at risk of unhealthy behavior is always of incredible value, not only in clinical healthcare practice, but from the point of view of a public health professional. The clinician will know with which patient profile he or she must take their time, and will be able to assess the possibility of problems such as abuse or dependence, even at early ages. Thus, he/she will be able to identify alcohol abuse or dependence or their risk in time, taking measures, seeking treatments and therapies at earlier stages, and improving, in this way, the prognosis of their patients. Health professionals and epidemiologists will be able to direct their messages in a more concrete way, knowing the population to which they are addressed, and therefore the messages will be more effective. Finally, higher alcohol consumption in certain minority groups, such as homosexual students, may be related to strategies of evasion, which could be addressed by promoting the acceptance of sexual diversity. In this line, recent studies have focused on protective factors such as resilience and coping with these risky patterns, specifically among homosexual women [70,71].

## Figures and Tables

**Table 1:** Characteristics of female students in the initial sample and in follow-up samples. Spit for Science, Cohort 1 and Cohort 2.

	Proportion of Subjects (%)				
Characteristics	Initial *n* = 3113	1-Year Follow-up *n* = 1667	2-Year Follow-up *n* = 1315	3-Year Follow-up *n* = 1112	*p*-Value
**Diagnostic and Statistical Manual of Mental Disorders, 4th Edition**					
Non- abuse and non-dependence	78.8	81.8	80.6	81.2	
Abuse	14.1	12.5	13.9	12.8	
Dependence	7.1	5.7	5.5	6.0	0.394
**Age of onset of alcohol use**					
Older than 18 years	19.9	27.1	31.5	32.2	
18 years	29.3	30.6	27.5	27.6	
17 years	21.1	18.2	17.2	16.1	
16 years	16.2	14.2	13.5	13.0	
Younger than 16 years	13.5	9.9	10.4	11.1	<0.001
**Cannabis consumption**					
No	66.0	67.5	69.1	69.6	
Yes	34.0	32.5	30.9	30.4	0.105/0.010 (lineal association)
**Sexual orientation**					
Heterosexual	89.7	90.2	90.6	90.3	
Homosexual	2.1	2.1	1.9	1.6	
Bisexual	8.2	7.6	7.6	8.1	0.915
**Ethnicity**					
White	48.9	43.7	42.7	42.5	
Asian	15.2	18.5	20.2	20.6	
Black	22.8	25.2	25.2	25.0	
Hispanic	5.9	5.1	5.6	5.9	
More than one race	6.7	7.0	6.7	6.0	<0.001
**Residence**					
Parental home	6.3	6.1	6.5	7.0	
Residence hall	88.6	89.7	88.9	88.6	
Off-campus housing	5.2	4.2	3.9	4.4	0.580
**Work status**					
Currently not working	72.9	73.8	74.7	74.1	
Part-time	24.4	23.1	22.0	22.4	
Full-time	2.7	3.1	3.3	3.5	0.628

**Table 2:** Characteristics of male students in the initial sample and in follow-up samples. Spit for Science, Cohort 1 and Cohort 2.

	Percentage of Subjects (%)				
Characteristics	Initial *n* = 2057	1-Year Follow-up *n* = 883	2-Year Follow-up *n* = 644	3-Year Follow-up *n* = 540	*p*-Value
**Diagnosis of alcohol disorder according to DSM-IV**					
Non- abuse and non- dependence	74.6	75.5	76.9	73.3	
Abuse	17.7	16.6	15.8	19.1	
Dependence	7.8	7.6	7.3	7.6	0.913
**Age of onset of alcohol use**					
Older than 18 years	16.5	23.0	26.5	27.1	
18 years	32.8	32.4	30.4	32.6	
17 years	22.2	20.5	19.9	19.1	
16 years	14.6	11.4	11.3	11.0	
Younger than 16 years	14.0	12.7	11.9	10.2	<0.001
**Cannabis consumption**					
No	56.9	57.7	60.7	62.9	
Yes	43.1	42.3	39.3	37.1	0.087/0.012 (linear association)
**Sexual orientation**					
Heterosexual	89.8	90.5	88.8	90.2	
Homosexual	7.1	6.7	7.8	6.2	
Bisexual	3.0	2.8	3.3	3.6	0.902
**Ethnicity**					
White	55.7	54.7	53.0	52.0	
Asian	17.7	20.9	21.8	21.8	
Black	14.9	14.6	16.7	16.3	
Hispanic	6.8	6.2	5.6	6.3	
More than one race	5.0	3.5	2.9	3.6	0.110
**Residence**					
Parental home	7.1	7.1	5.7	6.4	
Residence hall	85.8	88.4	89.2	88.1	
Off-campus housing	7.1	4.6	5.1	5.5	0.171
**Work status**					
Currently not working	76.7	75.6	76.5	74.9	
Part-time	21.3	22.7	22.6	24.2	
Full-time	2.0	1.7	1.0	0.9	0.418

**Table 3:** Rates of prevalence of Alcohol abuse and Alcohol Dependence among female participants. Spit for Science, Cohort 1 and Cohort 2.

	Proportion of Subjects (%)									
	Alcohol Abuse (DSM-IV)					Alcohol Dependence (DSM-IV)				
	Period					Period				
Participants Characteristics	First Year, Fall	First Year, Spring	Second Year, Spring	Third Year, Spring	Fourth Year, Spring	First Year, Fall	First Year, Spring	Second year, SPRING	Third Year, Spring	Fourth Year, Spring
**Age of onset of alcohol use**										
Older than 18 years	3.3	3.9	3.1	4.9	5.0	1.1	0.6	1.5	1.0	1.3
18 years	7.2	7.1	11.0	10.2	16.0	2.5	2.5	3.3	3.3	4.9
17 years	13.5	14.9	18.0	19.8	21.8	5.6	5.5	5.7	8.3	5.8
16 years	29.5	28.4	28.6	31.4	22.6	11.8	6.7	9.7	7.2	6.5
Younger than 16 years	46.4*	36.6*	31.5*	35.1*	46.7*	18.7*	11.0*	13.8*	8.8*	17.1*
**Ethnicity**										
White	29.9	18.6	19.0	19.6	23.0	9.2	4.7	6.6	4.9	7.5
Asian	12.7	10.9	11.7	11.3	9.1	8.1	5.2	6.7	4.3	3.4
Black	8.3	11.1	7.5	11.5	12.5	6.1	5.0	3.1	2.6	2.9
Hispanic	11.4	15.3	20.8	13.6	18.6	5.5	3.5	3.9	6.8	3.4
More than one race	19.8*	20.9*	17.3*	14.5^	13.3*	4.1	5.5	5.4	9.2	5.0
**Sexual orientation**										
Heterosexual	17.5	13.6	14.1	14.7	15.8	6.9	4.2	4.8	4.2	4.9
Homosexual	16.7	12.0	35.5	35.0	23.5	5.3	0	9.7	5.0	5.9
Bisexual	20.6	18.9	17.9*	18.5^	26.8	9.7	5.3	8.5	6.5	8.5
**Residence**										
Parental home	17.5	11.6	7.4	5.9	18.9	0.0	1.4	2.1	2.0	4.2
Residence hall	17.9	15.9	9.6	8.5	9.8	9.4	4.9	2.9	2.4	2.8
Off-campus housing	28.9	20.7	20.5*	20.2*	19.1^	16.7^	5.2	7.7	5.9^	6.2
**Work status**										
Currently not working	17.7	15.6	14.1	17.6	17.8	8.7	4.8	4.8	6.4	8.0
Part-time	21.6	15.4	14.5	13.4	15.6	10.4	4.6	5.3	2.9	3.9
Full-time	9.5	31.8	37.8*	26.4^	26.9	13.0	4.5	16.2^	11.3^	7.5
**Total**	18.4	15.8	15.0	15.4	17.0	7.8	4.7	5.3	4.5	5.3

* *p* < 0.01; ^ *p* < 0.05.

**Table 4:** Rates of prevalence of Alcohol abuse and Alcohol Dependence among male participants. Spit for Science, Cohort 1 and Cohort 2.

	Proportion of Subjects (%)									
	Alcohol Abuse (DSM-IV)					Alcohol Dependence (DSM-IV)				
	Period					Period				
Participants Characteristics	First Year, Fall	First Year, Spring	Second Year, Spring	Third Year, Spring	Fourth Year, Spring	First Year, Fall	First Year, Spring	Second Year, Spring	Third Year, Spring	Fourth Year, Spring
**Age of onset of alcohol use**										
Older than 18 years	9.7	8.3	7.1	5.1	8.1	6.3	2.1	2.4	4.3	3.3
18 years	7.1	16.5	14.1	20.2	22.5	3.3	4.5	6.2	7.5	11.9
17 years	23.8	22.8	27.2	29.7	34.1	3.3	6.1	9.5	8.1	15.9
16 years	35.4	33.0	34.6	34.9	34.6	11.2	12.2	16.0	17.5	23.1
Younger than 16 years	44.5*	39.2*	41.7*	28.6*	21.7*	19.2*	13.7*	11.9*	18.8*	10.9*
**Ethnicity**										
White	24.6	24.1	24.9	26.5	25.3	7.5	7.2	9.0	10.8	12.3
Asian	12.3	12.1	15.7	10.7	14.3	4.3	5.6	6.1	5.4	4.8
Black	12.3	20.0	10.5	10.2	20.8	7.7	2.7	6.3	5.7	16.7
Hispanic	16.2	19.6	19.0	10.0	17.2	7.1	12.5	7.1	12.9	13.8
More than one race	10.5 *	25.7	11.5*	50.0*	22.2	9.4	2.9	7.7	18.8	11.1
**Sexual orientation**										
Heterosexual	19.3	19.3	19.8	19.1	22.5	5.7	6.5	8.2	9.4	11.5
Homosexual	17.6	7.9	24.0	27.7	13.3	2.9	0.0	6.0	6.4	6.7
Bisexual	14.3	27.8	23.8	25.0	23.5	0.0	16.7	0.0	10.0	11.8
**Residence**										
Parental home	15.4	18.4	13.5	5.1	11.8	11.1	2.6	7.7	7.7	8.8
Residence hall	21.2	20.7	12.7	9.6	15.4	7.6	6.4	4.4	5.2	5.8
Off-campus housing	36.7^	31.2	24.7*	24.6*	23.7^	20.6^	10.4	9.4	10.2	12.4
**Work status**										
Currently not working	22.9	21.9	19.0	19.1	23.2	8.9	6.6	7.4	7.6	11.6
Part-time	19.6	20.0	23.7	21.3	19.8	7.1	5.7	7.8	11.0	11.5
Full-time	21.4	40.0	23.5	26.3	31.8	0.0	30.0^	11.8	10.5	9.1
**Total**	20.6	21.5	20.5	20.2	21.9	7.1	6.6	7.7	9.1	11.3

* *p* < 0.01; ^ *p* < 0.05.

**Table 5:** Explanatory models for alcohol abuse and alcohol dependence. Generalized Linear Mixed Models. Spit for Science, Cohort 1 and 2.

	Odds Ratio (95% Confidence Interval)			
	Females		Males	
Predictors	Alcohol Abuse	Alcohol Dependence	Alcohol Abuse	Alcohol Dependence
**Age of onset of alcohol use**				
Older than 18 years	1	1	1	1
18 years	3.01 (2.00–4.06)	2.83 (1.39–5.76)	2.24 (1.53–3.27)	1.83 (1.07–3.14)
17 years	5.33 (3.44–7.05)	5.66 (2.80–11.43)	4.15 (2.84–6.06)	2.40 (1.38–4.19)
16 years	10.19 (6.85–13.92)	7.95 (3.96–15.99)	6.62 (4.46–9.84)	5.57 (3.21–9.66)
Younger than 16 years	14.98 (9.53–19.92)	11.83 (5.89–23.78)	7.41 (4.95–11.10)	6.24 (3.58–10.88)
**Residence**				
Parental home	1	1		
Residence hall	0.96 (0.64– 1.44)	1.56 (0.67– 3.65)		
Off-campus housing	1.73 (1.16–2.56)	2.76 (1.19–6.40)		
**Ethnicity**				
Black/African American	1	1	1	1
Asian	1.18 (0.85–1.65)	1.24 (0.91–1.70)	0.84 (0.57–1.24)	0.68 (0.40–1.15)
Hispanic	1.22(0.81–1.84)	1.16 (0.78–1.72)	0.90 (0.57–1.42)	1.30 (0.73–2.29)
White	1.58 (1.23–2.02)	1.51 (1.19–2.91)	1.50 (1.11–2.03)	1.02 (0.68–1.52)
More than one race	1.73 (1.19–2.51)	1.73 (1.21–2.48)	1.35 (0.81–2.24)	1.19 (0.61–2.34)
**Sexual orientation**				
Heterosexual	1			
Homosexual	2.09 (1.21–3.58)			
Bisexual	1.28 (0.97–1.69)			
**Religiosity**				
Tercile 1 (Low)			1	1
Tercile 2 (Medium)			0.80 (0.65–0.96)	0.90 (0.68–1.19)
Tercile 3 (High)			0.58 (0.43–0.78)	0.76 (0.50–1.14)
**Work status**				
Currently not working	1	1		
Part-time	0.87 (0.72–1.04)	0.68 (0.50–0.92)		
Full-time	1.69 (1.14–2.52)	1.78 (1.00–3.16)		
**Period**				
First year, fall	1	1	1	1
First year, spring	0.92 (0.68–1.24)	0.44 (0.28–0.70)	1.22 (0.95–1.54)	0.89 (0.71–1.31)
Second year, spring	0.80 (0.58–1.10)	0.44 (0.27–0.74)	1.24 (0.95–1.62)	1.16 (0.78–1.72)
Third year, spring	0.85 (0.60–1.20)	0.39 (0.23–0.67)	1.36 (1.02–1.80)	1.43 (0.95–2.16)
Fourth year, spring	0.96 (0.67–1.39)	0.46 (0.26–0.82)	1.54 (1.13–2.11)	1.98 (1.33– 2.95)

* Multivariate models adjusted only by the variables included in the column.

**Table 6:** Early age of onset of alcohol use population; attributable fractions for alcohol abuse and alcohol dependence. Spit for Science, Cohort 1 and 2.

	Proportion (%) of alcohol abuse removed if…				Proportion (%) of alcohol dependence removed if…			
	…one third started alcohol use one year later	…one half started alcohol use one year later	…all students started alcohol use one year later	…all students started e alcohol use after age 18 years	…one third started alcohol use one year later	…one half started alcohol use one year later	…all students started alcohol use one year later	…all students started alcohol use after age 18 years
Females	14.59	22.11	44.21	80.9	16.15	24.46	48.93	71.7
Males	18.06	27.37	54.74	71.0	16.40	24.85	49.69	63.5
